# The Adverse Effects of Heavy Metals with and without Noise Exposure on the Human Peripheral and Central Auditory System: A Literature Review

**DOI:** 10.3390/ijerph13121223

**Published:** 2016-12-09

**Authors:** Marie-Josée Castellanos, Adrian Fuente

**Affiliations:** École d’orthophonie et d’audiologie, Faculté de Médecine, Université de Montréal, Montreal, QC H3C 3J7, Canada; castellanos1011@hotmail.com

**Keywords:** auditory brainstem response, hearing loss, heavy metals, humans, noise, pure-tone audiometry

## Abstract

Exposure to some chemicals in the workplace can lead to occupational chemical-induced hearing loss. Attention has mainly focused on the adverse auditory effects of solvents. However, other chemicals such as heavy metals have been also identified as ototoxic agents. The aim of this work was to review the current scientific knowledge about the adverse auditory effects of heavy metal exposure with and without co-exposure to noise in humans. PubMed and Medline were accessed to find suitable articles. A total of 49 articles met the inclusion criteria. Results from the review showed that no evidence about the ototoxic effects in humans of manganese is available. Contradictory results have been found for arsenic, lead and mercury as well as for the possible interaction between heavy metals and noise. All studies found in this review have found that exposure to cadmium and mixtures of heavy metals induce auditory dysfunction. Most of the studies investigating the adverse auditory effects of heavy metals in humans have investigated human populations exposed to lead. Some of these studies suggest peripheral and central auditory dysfunction induced by lead exposure. It is concluded that further evidence from human studies about the adverse auditory effects of heavy metal exposure is still required. Despite this issue, audiologists and other hearing health care professionals should be aware of the possible auditory effects of heavy metals.

## 1. Introduction

Heavy metals such as lead, mercury and arsenic are present in the environment in many forms: as a soil component, water contaminant, dust particle in urban areas [1], and in many others. Villages located around battery recycling centers in the Andes mountains [2], homes containing lead-based paint in the United States [3], some Inuit villages [4] and children living near some industrial areas in Poland [5] are examples of reported populations non occupationally exposed to these agents. In addition, heavy metals are widely present in a number of industrial sectors including mining and construction and millions of workers are potentially exposed to these agents in the workplace [6].

It has been consistently shown that low as well as high exposure levels to heavy metals have an adverse effect on human health, leading to conditions such as cardiovascular and pulmonary dysfunctions [7,8]. In addition, some studies have shown an association between exposure to heavy metals and hearing disorders in animals [9,10]. Apostoli et al. [9] demonstrated that high doses of cobalt induced optic and auditory neuropathy in rabbits. Wu et al. [11] showed that lead toxicity had an adverse impact on the peripheral fibers of the auditory nerve. In addition, abnormalities in auditory brainstem response (ABR) latencies induced by lead exposure have been shown to occur in rhesus monkeys [12]. Similarly, Ozcaglar et al. [13] found in rats an adverse effect of cadmium exposure on ABR wave I latency. Other studies have found an effect of heavy metals on outer hair cells in experimental animals by using otoacoustic emissions (OAEs) [12,13]. Thus, exposure to heavy metals seems to adversely affect the peripheral and central auditory system in experimental animals.

Regarding the effect of heavy metals on the human auditory system, a recent report [14] has linked Beethoven’s progressive hearing loss to axonal degeneration due to a continuous exposure to lead. Despite the growing interest from researchers and clinicians about the auditory effects induced by chemical exposure, not much attention has been specifically given to the effects of heavy metals. Therefore, the aim of this manuscript is to review the scientific evidence about the adverse effects of heavy metal exposure on the human auditory system. Studies investigating both occupational and non-occupational exposure to heavy metals have been included in this literature review.

## 2. Methodology

**Figure 1:**
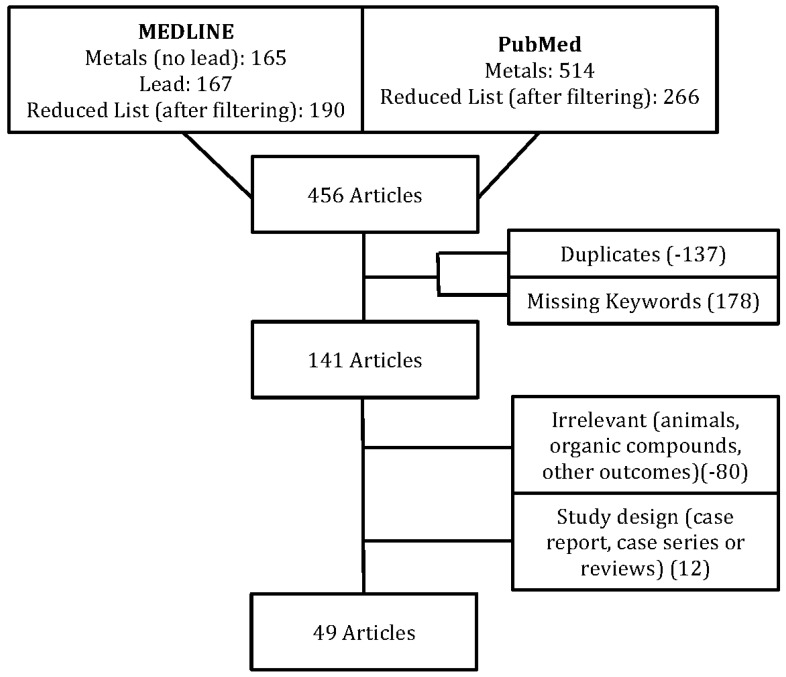
Methodological strategy for article selection.

The specific heavy metals to be investigated were chosen based on their hazardous level to humans. To establish this list, the ten most hazardous heavy metals to humans were selected from the Priority List of Hazardous Substances given by the Agency for Toxic Substances and Disease Registry (ATSDR) [15] which has been used as a reference for other studies as well as for establishing environmental guidelines [16]. A set of criteria was established to select relevant articles. These criteria were composed of the exclusion of articles investigating organic compounds or animals. Additional criteria concerning language and study design were also established such as articles not available in English, case reports, case series and reviews were excluded. The literature search was then done on 26 February 2016, using two databases: MEDLINE and PubMed. The search in MEDLINE was done with the following combination of keywords: (*heavy metals OR arsenic OR cadmium OR chromium OR cobalt OR copper OR manganese OR mercury OR nickel OR zinc*) *AND* (*toxicity OR poisoning OR ototoxicity*) *AND* (*hearing OR auditory*), which resulted in 165 articles. The keyword “*lead*” was excluded as with its homograph “*lead*”, the search resulted in numerous irrelevant results. A second search was then done for articles containing (*lead poisoning OR lead toxicity OR lead*) in their title AND (*hearing OR auditory*) as keywords, resulting in 167 articles. The MEDLINE searches yielded a combined 321 unique articles. Filtering by “humans” and “English language”, this list was reduced to 190 articles. The literature search done in PubMed used the following strategy: ((*heavy metals OR arsenic OR cadmium OR chromium OR cobalt OR copper OR lead OR manganese OR mercury Or nickel Or zinc*) *AND* (*toxicity OR poisoning OR ototoxicity*)) *AND* (*hearing OR auditory*). This search resulted in 514 articles. Filtering the studies by “humans” and “English language” reduced the list to 266 articles. After combining the two lists, deletion of duplicates (137) and articles missing the captioned keywords (178), a total of 141 articles remained. The abstracts of these 141 articles were retrieved and analyzed. Articles were further filtered by applying the criteria mentioned above (exclusion of organic compounds, animals or irrelevant articles, case reports, case series and reviews), leaving 49 articles as the final list. Figure 1 summarizes the method mentioned above.

In order to review articles investigating the possible interaction in humans between heavy metal and noise exposure on hearing thresholds a separate search was carried out. This literature search was conducted using Pubmed with the advanced search builder. The following strategy was used, ((*heavy metals*) AND *noise*) AND *hearing loss*. A total of 53 entries were obtained. Titles and abstracts (when available) for all 53 entries were reviewed. The full articles of 5 entries were accessed and finally only three articles were selected as they did investigate a possible interaction between heavy metals and noise on hearing thresholds. 

## 3. Effects of Heavy Metals on the Human Auditory System

None of the studies found investigated the effects of chromium, cobalt, copper, nickel or zinc exposure on the human auditory system.

### 3.1. Arsenic

Populated areas contaminated with arsenic include those near copper smelters [17], regions with cross-contaminated groundwater used for agriculture and drinking [18,19] and regions near gold mines [20,21]. All five studies mentioned below have investigated non-occupational populations exposed to arsenic due to environmental pollution. No studies investigating the possible auditory effects of arsenic exposure in the workplace were found in the literature. See Table 1 for a summary of the studies discussed below. 

Bencko and Symon [22] investigated a group of fifty-six 10-year-old children in Czechoslovakia (a former central European country) living near a power plant that burned coal with a high concentration of arsenic. A non-exposed control group of 51 children of the same age as the exposed group was also investigated. Air-conduction hearing thresholds (125–8000 Hz) along with bone-conduction thresholds (125–4000 Hz) were obtained. Results showed that arsenic-exposed children presented with an average for air-conduction pure-tone thresholds (125–8000 Hz) 2.7 dB higher than non-exposed children. In addition, they presented significantly higher (i.e., worse) pure-tone thresholds than non-exposed children for 125 (*p* = 0.0005), 250 (*p* = 0.001), 500 (*p* = 0.05), 1000 (*p* = 0.05) and 8000 (*p* = 0.01) Hz. Contrasting results were found by Milham [23]. The author reported that 1.4% of school-age children living near a copper smelter facility in Tacoma (Washington, DC, USA) failed the hearing screening for the years 1967–1968. The failure prevalence for all schools in the city was 5.7%. Thus, children who lived and attended a school near the copper smelter did not present with a higher prevalence of hearing loss than the general population of the same age in the city (the *p*-value was not reported).

In an adult population, Supapong and Sriratanabun [24] investigated a group of 29 female subjects with a history of arsenic exposure along with 27 non-exposed control female subjects in Thailand. Results for arsenic-exposed subjects showed 1.59, 3.58, and 5.37 ms for ABR absolute latencies of waves I, III and V, respectively. Results for non-exposed controls were 1.58, 3.48, and 5.28 ms for absolute latencies of waves I, III and V, respectfully. Differences between groups for the ABR absolute latencies were not statistically significant (*p* = 0.81, *p* = 0.09, and *p* = 0.12, for waves I, III and V, respectively). Similarly, no significant differences between groups were found for I–III (*p* = 0.06), III–V (*p* = 0.92), and I–V (*p* = 0.12) inter-peak latencies (IPLs).

Further evidence about the adverse effects of arsenic on auditory function in adults is provided by the study conducted by Guo et al. [18] in Inner Mongolia (Mainland China). The authors investigated 680 subjects living and working in villages affected by arsenic contaminated water and 189 control subjects living in villages without arsenic contamination in the water. The authors reported that 5.8% of villagers affected by arsenic contaminated water presented with hearing loss compared to 1.06% of control subjects (*p* = 0.005). It should be noted that the authors did not control for confounding variables such as age and noise exposure. In addition, the authors did not provide details about the definition of hearing loss they used in terms of audiometric frequencies and associated losses in decibels.

Contrasting results were observed by Shargorodsky et al. [25] in a cross-sectional analysis between urinary arsenic level and hearing loss in 875 adolescents aged between 12 and 19 years in the 2005–2008 National Health and Nutrition Examination Survey in the United States. The low frequency pure-tone average (PTA) (LPTA, 500, 1000 and 2000 Hz) and high frequency PTA (HPTA, 3000, 4000, 6000 and 8000 Hz) were calculated. Hearing loss was defined as a LPTA or HPTA greater than 15 dB HL in either ear. Results showed no association between urinary arsenic levels and hearing loss at either LPTA or HPTA. The reported odds ratios (OR) for LPTA and HPTA hearing loss for adolescents with the 4th highest quartile of urinary arsenic level as compared with adolescents with the 1st lowest quartile of urinary arsenic level were 0.87 (95% CI 0.41–1.85) and 0.94 (95% CI 0.51–1.72), respectively. 

### 3.2. Cadmium

Main sources of human exposure to cadmium are dietary intake [26,27], tobacco smoking habits [28] and air pollution [29]. Three studies about the auditory effects associated with cadmium exposure in the general population are presented below. See Table 1 for a summary of the studies discussed below. 

Thatcher et al. [30] carried out long latency auditory evoked potentials (long latency AEP) in 149 children aged between five and 16 years from the general population in Maryland (USA) and determined their cadmium and lead exposure levels. AEP were obtained from three scalp sites (central, parietal and occipital). Results showed a significant association between cadmium level in hair and the amplitude of the negative component of the long latency AEP at the central region of the scalp (*p* < 0.05). 

Choi et al. [31] investigated the possible association between the levels of cadmium and lead in blood and hearing levels in the U.S. general population. A database of 3698 adults aged 20 to 60 years was studied. Hearing loss was defined as PTA (500, 1000, 2000 and 4000 Hz) above (i.e., worse than) 25 dB HL in either ear. Subjects in the highest blood cadmium quintile had 13.8% (95% CI 4.6%–23.8%) higher hearing thresholds than did those in the lowest quintile when the model was adjusted by all confounding variables (*p* = 0.005). In addition, a doubling of blood cadmium was associated with a 4.1% (95% CI 1.2%–7.1%) increase in hearing thresholds. The fully adjusted OR for hearing loss comparing the highest versus the lowest blood cadmium quintiles was 1.7 (95% CI 1.1–2.7). The authors concluded that low level exposure to cadmium and lead may be important risk factors for hearing loss. 

Similar results were found by Shargorodsky et al. [25] who investigated 878 subjects selected from the 2005–2008 National Health and Nutrition Examination Survey. The results showed no significant association between blood cadmium level and hearing loss at any frequency. However, a significantly increased OR for the 4th quartile of blood cadmium levels (OR: 3.08, 95% CI 1.02–9.25) and low frequency hearing loss was found. A significant dose–response relationship was not observed (*p* = 0.13).

### 3.3. Lead

Lead poisoning is one of the most common metal poisonings [32]. Lead is present in many man-made structures, work environments and various products. Occupational lead exposure is often originated in work environments such as steel plants [33,34,35], glass factories [36,37,38], and other industries using lead-based products or processes [39,40]. Non-occupational lead exposure is often caused by the use of gasoline with lead additives [40,41,42], incineration of lead-containing waste [43,44], lead-based paints used in homes [3,45], ceramic glazes containing lead [46,47,48], electronic waste [44,49], other industrial lead-based product factories [50], lead-containing water networks [51] and contaminated food chains [52,53]. Most of the human studies about the effects of heavy metals on the auditory system have investigated the effects of lead. Therefore, most of the results from this literature review can be found in this section. A summary of these studies, clustered based on the population and/or auditory outcome used, is shown below. Table 2 and Table 3 summarize the studies investigating the auditory effects of lead exposure in adulthood and childhood, respectively. 

#### 3.3.1. Hearing Thresholds and Occupational Lead Exposure

Baloh et al. [54] were among the first authors to report hearing outcomes in occupationally lead-exposed workers. The authors investigated a group of 64 workers from a lead smelter and a control group of 31 workers without lead exposure. Results showed very similar pure-tone thresholds (250–8000 Hz) between both groups and thus no significant differences were found (*p*-value was not reported).

Opposite results were found by Farahat et al. [55]. The authors investigated 40 workers from a printing facility who were exposed to lead and 45 non-exposed control workers. Average pure-tone thresholds for 2000, 4000 and 8000 Hz among lead-exposed workers were significantly higher (*p* < 0.05) by 4.11 dB, 2.89 dB, and 4.18 dB, respectively, than non-exposed workers. In addition, workers with blood lead levels equal to or higher than 30 micrograms (µg)/deciliter (dL) (*n* = 22) presented with an average pure-tone threshold at 8000 Hz 3.14 dB significantly (*p* < 0.05) higher than non-exposed workers (*n* = 23). Similarly, among lead-exposed workers, those who were exposed for 10 or more years (*n* = 28) showed a significantly (*p* < 0.01) higher pure-tone threshold by 4.01 dB at 8000 Hz than lead-exposed workers who were exposed for less than 10 years (*n* = 17). 

Forst et al. [56] investigated the association between lead exposure (as measured in blood lead levels) and hearing thresholds. For such purpose, a cross-sectional, computerized dataset was accessed from a private occupational health screening company in the U.S. Audiometric data and blood lead level results were available for 183 workers. Hearing thresholds for each single frequency (500–6000 Hz) were categorized as normal (equal or lower than 10 dB HL) or abnormal (higher than 10 dB HL). Blood lead level was treated as a continuous variable. A significant correlation between blood lead level and abnormal hearing threshold was found only at 4000 Hz (*p* = 0.03). In addition, a non-significant trend (*p* > 0.05) was observed in which people with higher blood lead level presented with a higher percentage of abnormal hearing thresholds, especially at 3000 and 4000 Hz. 

Similar results were found by Wu et al. [39]. The authors conducted a cross-sectional study in two lead-battery manufacturing factories in Taiwan with the aim to determine possible independent and synergistic effects of lead and noise on hearing function. Blood lead level, environmental lead concentration, noise exposure level and hearing thresholds (500–8000 Hz) were obtained in 220 workers (mean age 36.7). Hearing function was defined as the pure-tone threshold at 4000 Hz in the worse ear. A significant (*p* < 0.05) dose–response relationship in both male and female workers between blood lead level and hearing level was observed with the exception of the lowest blood lead level. For example, male workers with blood lead levels of 25–40, 41–60, and above 60 µg/dL had a mean pure-tone threshold (at 4000 Hz) of 26.3 (standard deviation (SD) 19.1), 29.6 (SD 20.0), and 38.1 (SD 28.5), respectively. In addition, multiple linear regression analyses were carried out to determine the possible association between lead exposure, noise exposure and hearing ability. Lead exposure was evaluated as the blood lead level to indicate short-term lead exposure levels (model 1), and as the long-term exposure index (containing environmental lead concentration and duration of employment) to indicate long-term lead exposure levels (model 2). The noise exposure level (NEL) was obtained from an 8-h noise dosimetry. The results showed a significant association (regression coefficient = 0.02, *p* < 0.01) between the long-term lead exposure index (model 2) and decreased hearing ability, after adjusting for confounding variables (*p* < 0.01). Short-term lead exposure level and hearing function (model 1) were not significantly associated (*p* = 0.09). NEL was not significantly associated with hearing ability in either model (*p* = 0.46 for model 1, and *p* = 0.77 for model 2). In addition, a possible synergistic effect between lead and noise exposure was further explored in separate regression models. For this, interaction terms between NEL and the long-term exposure index, as well as NEL and the blood lead level were investigated. Results showed a non-significant association between these interaction terms and hearing ability at 4000 Hz (*p* > 0.05).

Similarly, Counter and Buchanan [57] investigated 30 adults chronically exposed to lead from ceramic-glazing work in the Andes Mountains in Ecuador. Subjects presented with a mean blood lead level above the World Health Organization health-based biological limits. Mean PTA (2000–8000 Hz) was obtained for the right and left ears separately for male and female workers. The mean PTA among male workers showed sensorineural hearing loss for the right (mean PTA = 29.5 dB HL) and left ear (37.5 dB HL). They had significantly poorer high frequency hearing sensitivity (*p* < 0.05) than female workers at 4000, 6000 and 8000 Hz in the right ear, and at 3000, 4000, 6000 and 8000 Hz in the left ear. Bivariate regression models between pure-tone thresholds at 3000, 4000, 6000 and 8000 Hz in the right and left ears, separately, and blood lead level for the whole group of participants (*n* = 30) were obtained. No significant associations between blood lead level and right ear pure-tone thresholds at 3000 (r = 0.056, *p* = 0.77), 4000 (r = 0.004, *p* = 0.98), 6000 (r = 0.034, *p* = 0.85), and 8000 Hz (r = 0.12, *p* = 0.52) were found. Similarly, left ear pure-tone thresholds at 3000 (r = 0.052, *p* = 0.78), 4000 (r = 0.011, *p* = 0.95), 6000 (r = 0.10, *p* = 0.57), and 8000 Hz (r = 0.164, *p* = 0.28) were not significantly associated with blood lead level. In addition, ABR was obtained from 12 workers who presented with a blood lead level between 18 and 80 µg/dL. Group mean of absolute latencies for waves I, III and V as well as I–III, I–V, and III–V IPL were within normal ranges. However, when subjects were divided according to their blood lead level, a dose–response relationship between absolute latency for waves I, III and V and blood lead levels was observed, according to the authors. The same was true between I–III, I–V, and III–V IPL and blood lead levels (no statistical test was applied by the authors and thus no p-value was reported).

Similar results were found in Taiwan by Hwang et al. [35]. The authors investigated a total of 412 steel plant workers. Pure-tone audiometry was bilaterally conducted at 500, 1000, 2000, 3000, 4000, 6000 and 8000 Hz. Blood levels for manganese, copper, zinc, arsenic, cadmium and lead were determined. The results showed that the blood lead level was significantly correlated with hearing thresholds at 500 (correlation coefficient (CC) = 0.13, *p* < 0.05), 2000 (CC = 0.19, *p* < 0.005), 3000 (CC = 0.21, *p* < 0.0005), 4000 (CC = 0.24, *p* < 0.0005), 6000 (CC = 0.23, *p* < 0.0005), and 8000 Hz (CC = 0.22, *p* < 0.0005). In addition, hearing thresholds among workers with a low (equal or lower than 4 µg/dL), medium (between 4 and 7 µg/dL) and high (higher than 7 µg/dL) blood lead level were compared. Workers with high blood lead levels presented with significantly worse hearing thresholds for most of the frequencies than workers with low and medium blood lead levels. Logistic regression analyses showed that high blood lead level was significantly associated with hearing loss (hearing thresholds equal or higher than 25 dB HL) at 3000 (OR = 4.49, 95% CI: 1.28–15.8), 4000 (OR = 6.26, 95% CI: 2.35–16.6), 6000 (OR = 3.06, 95% CI: 1.27–7.39) and 8000 Hz (OR= 6.16, 95% CI: 1.59–23.9). Important to mention is that noise exposure level above 80 dB was not significantly associated with any of the hearing thresholds at the explored frequencies.

#### 3.3.2. Hearing Thresholds and Non-Occupational Lead Exposure in Adulthood

Park et al. [58] conducted a study to investigate the possible association between chronic non-occupational lead exposure and risk for age-related hearing loss in the general population in the U.S. Lead levels in both cortical bone (tibia) and trabecular bone (patella) were obtained along with pure-tone thresholds (500–8000 Hz) in a community-based cohort of 448 men with a median follow-up of 23 years. The mean age of the group was 42.5 years at the time of the first audiometric evaluation. For analysis purposes, the better hearing threshold between the left and right ear was used for each frequency. The results showed that cortical and trabecular bone lead levels were significantly (*p* < 0.05) associated with pure-tone thresholds at 2, 3, and 4 kHz as well as with PTA (500–4000 Hz). In addition, inter-quartile range increases in cortical lead (15 µg/g) and trabecular lead (21 µg/g) were associated with 2.18 (95% CI 0.13–4.23) dB HL and 3.43 (95% CI 1.46–5.41) dB HL increases in hearing threshold at 4 kHz, respectively. The authors concluded that lead exposure may be an important risk factor for age-related hearing loss. 

Choi et al. [31] also investigated the effect of blood lead level on hearing thresholds in the U.S. general population. Subjects in the highest blood lead level quintile (2.80–54 µg/dL) had 18.6% (95% CI 7.4%–31.1%) higher hearing thresholds than did those in the lowest quintile (0.2–0.8 µg/dL). A doubling of blood lead level was associated with a 5.4% (95% CI 2.1%–8.8%) increase in hearing thresholds. A fully adjusted OR for hearing loss (PTA (500–4000 Hz) higher than 25 dB HL) of 1.4 (95% CI 0.8–2.5) was found when comparing the highest versus the lowest blood lead level quintiles. Importantly, the authors estimated the combined effect of exposures to lead and cadmium on hearing thresholds. Subjects with both high lead and cadmium blood levels had a 19% (95% CI 9.7%–29.1%) increase in PTA (500–4000) as compared with subjects with both low blood lead and cadmium levels. The authors concluded that this increase in PTA was consistent with additive effects of co-exposure to lead and cadmium.

In addition, Shargorodsky et al. [25] investigated 2535 subjects who were selected from the 2005–2008 National Health and Nutrition Examination Survey in the U.S. The results showed an association between the quartile with the highest level of lead exposure and a higher high-frequency PTA (OR: 2.22; 95% CI 1.39–3.56). 

#### 3.3.3. ABR and Occupational Lead Exposure

Discalzi et al. [59] investigated a group of 49 lead-exposed workers and a non-exposed control group of 49 age- and gender-matched subjects. Blood lead levels were determined along with ABR. I–III, I–V and III–V IPL were compared between groups. Lead exposed workers presented with significantly longer absolute wave latency and IPL than non-exposed subjects. For example, lead-exposed subjects presented an absolute wave V latency 0.18 ms (95% CI 0.11–0.25) longer than non-exposed subjects (*p* = 0.000) and an I–V IPL 0.11 ms (95% CI 0.07–0.16) longer than non-exposed subjects. In addition, lead-exposed workers with blood lead levels above 50 µg/dL presented with an I–V IPL 0.08 ms longer (95% CI 0.003–0.16) than lead-exposed workers with blood lead levels below 50 µg/dL (*p* = 0.04). 

Bleecker et al. [60] conducted ABR on a group of 359 male, lead smelter workers with a mean age of 41 years. ABR data obtained in the right ear were used for the analyses. Significant correlations adjusted for age between the cumulative lead dose represented by the working-life time integrated blood level (IBL) index and ABR wave I (r = 0.13, *p* < 0.01) and wave III (r = 0.16, *p* < 0.01) latencies were found. In addition, the working-lifetime weighted average blood lead level was significantly associated with ABR wave III latency. In addition, multivariate regression analyses showed that IBL significantly contributed to the variance of both waves III latency (*p* = 0.000) and I–III IPL (*p* < 0.03). 

In addition, Discalzi et al. [61] investigated ABR I-V IPL in 22 workers exposed to lead in a battery factory along with 22 age- and gender-matched subjects. I–V IPL was 0.15 (95% CI 0.07–0.24) ms longer in workers exposed to lead as compared to non-exposed control subjects (*p* = 0.001). In addition, lead-exposed workers were divided into two groups based on their blood lead level. The I–V IPL was 0.12 ms longer in workers with blood lead levels above 50 µg/dL than in workers with blood lead levels below 50 µg/dL (*p* = 0.034). In addition, Lille et al. [62] investigated the ABR in 13 patients with a history of occupational exposure to lead. Results showed that for only one patient ABR results were abnormal, showing an I–V IPL of 4.7 ms. 

Opposite results were found by Murata et al. [37] in China. The authors investigated the ABR in a group of 36 female workers from a glass factory exposed to lead and a control group of 15 non-exposed female textile workers. ABR was conducted for the right ear in all subjects. Possible significant differences between groups were investigated for ABR wave I latency as well as I–III and I–V IPL. No significant differences between groups (*p* > 0.05) for the aforementioned ABR results were found when controlling for age. In addition, a correlation between blood lead level and ABR results (as mentioned above) was not observed (*p* > 0.05). Also in China, similar results were found by Yokoyama et al. [38]. The authors investigated 29 female workers (aged 21–30 years) from a glass factory exposed to lead and a non-exposed control group of 14 female workers (aged 22–29 years) from a textile factory. The ABR was conducted in both groups. Wave I latency as well as I–III and III–V IPL were compared between groups. Results for waves I, I–III IPL and III–V IPL were 1.66 (1.42–1.84), 2.07 (1.69–2.41) and 1.78 (1.56–2.08) ms for lead-exposed workers, respectfully, as compared with 1.70 (1.49–2.08), 2.04 (1.78–2.38) and 1.75 (1.56–1.96) ms for non-exposed subjects. Differences were not statistically significant (*p* > 0.05). Thus, results from the latter two studies do not provide evidence about the adverse effects of lead exposure on either hearing sensitivity (ABR wave I) or on the conduction of auditory information at the brainstem level (I–III, I–V and III–V IPL). However, it should be taken into consideration that the sample sizes were rather small as compared to Bleecker et al. [60] who found significant correlations between blood lead levels and ABR results. Another important variable that may have influenced such differences is gender. Murata et al. [37] and Yokoyama et al. [38] investigated only female workers, whereas Bleecker et al. [60] investigated only male workers. 

#### 3.3.4. ABR and Non-Occupational Lead Exposure in Adulthood

Among the first reports about lead exposure and ABR comes from Holdstein et al. [63]. The authors investigated a group of 29 adults and children who were accidentally exposed to lead in food for around one to two years. The study was conducted one year after lead exposure was detected. All subjects were asymptomatic but presented with high blood lead levels. Their mean age was 27 years old (range of 8–56). Pure-tone audiometry, speech audiometry and ABR were conducted in all subjects. For ABR, two rates were used, 10 stimuli per second (10/s) and 55 stimuli per second (55/s). ABR I–III, I–V and III–V IPL were obtained. In addition, the difference for each IPL between both presentation rates was determined. Results showed that subjects with higher blood lead levels presented with significantly longer I–III IPL than those with low blood lead levels (*p* < 0.025 at 10/s; *p* < 0.05 at 55/s). Pure-tone thresholds and speech perception scores were not reported by the authors.

#### 3.3.5. Lead-Induced Auditory Dysfunction during Childhood

Otto et al. [64] were some of the first authors to investigate the effects of lead on the auditory system in children. A total of 49 children ranging in age between 6 and 12 years were selected. Sources of lead exposure included lead dust from the clothing of parents who worked in battery factories or peeling lead paint in children’s homes. ABR was conducted in all children. The results showed a significant effect of original blood lead levels (previously obtained when children were suspected to be exposed to lead) on ABR waves III (r^2^ = 0.21) and V (r^2^ = 0.16) latencies. These latencies increased linearly with original blood lead levels. Further evidence about lead exposure and auditory dysfunction in children is provided by Osman et al. [5]. The authors investigated a group of 286 children from three towns in the Katowice industrial region in Poland. Based on blood lead levels, the children were divided into two groups, low (below 50 μg/L) and high (above 150 μg/L) blood lead levels. Pure-tone thresholds were obtained at 500, 1000, 2000, 4000, 6000 and 8000 Hz. ABR was also conducted. Results showed that hearing thresholds at all tested frequencies significantly increased with increasing blood lead levels (*p* = 0.003 for 6 kHz in the left ear; *p* < 0.001 at all the other frequencies), therefore suggesting that children with high blood lead levels have significantly higher hearing thresholds than children with low blood lead levels. Children with the highest blood lead levels presented with a significantly increased latency for ABR wave I (B = 0.057, 95% CI = 0.016–0.098) as compared to children with lowest blood lead levels. Similar results were found by Abdel Rasoul et al. [65]. The authors investigated the association between blood lead level and hearing function in 190 primary school children living in urban and rural areas in Egypt. Environmental lead levels were significantly higher in urban schools than in rural schools (*p* < 0.001) and blood lead levels were significantly correlated with pure-tone thresholds (*p* = 0.03). Schwartz and Otto [66] investigated the association between blood lead level and hearing thresholds in 4519 children and youth who were part of the Second National Health and Nutrition Examination Survey (NHANES II) in the United States. The results showed that blood lead levels were significantly associated (*p* < 0.00001) with increased right and left hearing thresholds at 500, 1000, 2000 and 4000 Hz. Similarly, Schwartz and Otto [67] found a significant association between blood lead levels and pure-tone thresholds at 500 Hz (*p* < 0.001), 1000 Hz (*p* < 0.001), 2000 Hz (*p* < 0.001) and 4000 Hz (*p* = 0.017) among Hispanic children aged 6–19 years. In addition, Kamel et al. [68] in Egypt found a significant positive correlation between blood lead level and PTA (r = 0.21, *p* < 0.001) in a group of 250 children aged between eight and 10 years. 

Baumann et al. [69] investigated the association between blood lead level and long latency AEP in 49 children from low socioeconomic families. The long latency AEP were obtained from electrodes at Cz and Pz locations from a target (100-Hz tone) and non-target (500-Hz tone) stimulus. The target stimulus was presented 25% of the trials. Results showed a significant association between blood lead level and the positive peak from the long latency AEP obtained from the non-target stimulus at Cz (*p* = 0.017) and Pz (*p* = 0.001). 

Results presented by Zou et al. [70] support the above correlations. The authors conducted a study in 114 children (aged one to six years) with the aim to determine whether lead exposure in children affects ABR results. Blood lead levels were determined and children were divided into two groups: low (below 100 μg/L) and high (equal to or above 100 μg/L) lead levels. Peak-latencies for waves I (left ear: *p* < 0.001; right ear: *p* = 0.038) and V (left ear: *p* = 0.012; right ear: *p* = 0.018) were significantly longer in both ears in children with high blood lead levels as compared to children with low blood lead levels. In addition, children with high blood lead levels had a significantly longer wave III latency in the left ear (*p* = 0.026). No significant differences for wave III absolute latency for the right ear were found (*p* = 0.107). In addition, significant positive correlations between peak-latencies in both ears and blood lead levels were found for waves I (left ear: r = 0.508; *p* < 0.001, right ear: r = 0.365, *p* < 0.001) and IV (left ear: r = 0.262, *p* < 0.005; right ear: r = 0.247, *p* < 0.009), after controlling for confounders. The absolute latency for wave III was significantly correlated with blood lead levels only in the left ear (r = 0.207, *p* = 0.029). Prolonged latencies for all waves, including wave I, suggest a cochlear dysfunction rather than retrocochlear dysfunction.

Based on the aforementioned studies, lead exposure in children may be associated with higher (i.e., worse) hearing thresholds and this may relate to lead-induced cochlear dysfunction. However, other studies have failed to find an association between lead exposure in children and auditory dysfunction, as explained below.

A number of studies have been conducted in lead-exposed children from the Andes Mountains in Ecuador. Counter et al. [71] investigated children living in Andean villages of Ecuador with high lead contamination from discarded automobile batteries used in the local ceramic glazing industry. A total of 107 children living in the lead glazing area along with 39 children living in a geographically distant area were investigated. ABR results in children exposed to lead showed normal results, and no correlation between blood lead levels and ABR IPL was found (*p* > 0.05 at all wave peaks and IPLs). In addition, no correlation between blood lead levels and pure-tone thresholds was found (no *p*-value reported). In addition, with a different sample, Counter et al. [72] investigated 62 Andean school children living in a lead contaminated area of Ecuador and 12 children living in a neighboring gold mining area without known lead exposure. Lead-exposed children presented with normal pure-tone thresholds (250–8000 Hz) as well as normal ABR absolute latencies and IPL.

In another study, Counter [73] investigated 112 Andean lead-exposed children. Results showed no significant association between blood lead levels and ABR I–III (*p* = 0.93), II–V (*p* = 0.16), I–V (*p* = 0.35), and I–VI IPL (*p* = 0.27). However, the author reported that some children presented with prolonged ABR IPL. Counter et al. [74] also investigated 66 children from two to 15 years of age living in lead contaminated villages in the Ecuadorian Andes Mountains. The association between lead exposure, presence of anemia and the ABR was investigated. Results showed that the children as a group presented with hemoglobin levels that suggest anemia in addition to blood lead levels indicative of lead poisoning. A negative significant correlation between ABR absolute latencies and hemoglobin levels was found. In addition, children with low hemoglobin levels (<11 g/L) showed significantly prolonged absolute latencies for ABR waves II (r = −0.351, *p* = 0.005), III (r = −0.301, *p* = 0.015), IV (r = −0.365, *p* = 0.004) and V (r = −0.298; *p* = 0.016) compared to children with normal hemoglobin levels. However, no significant association was shown between hemoglobin level and the absolute latency of wave I (r = −0.213, *p* = 0.085). Despite finding a significant association between hemoglobin levels and ABR wave latencies, no significant association between the latter and blood lead levels was found (Z = −0.400, *p* = 0.689).

Auditory outcomes other than pure-tone audiometry and ABR have also been used in pediatric populations exposed to lead. Buchanan et al. [75] measured distortion product OAE (DPOAE) in 14 children and five adults who were living in highly lead contaminated areas in the Andes Mountains of Ecuador. Results showed no hearing loss and no correlation between blood lead levels and DPOAE amplitudes (no *p*-value reported). Similar results were found by Buchanan et al. [2]. The authors measured DPOAE in a total of 53 children aged between six and 16 years. All children were living in lead contaminated environments in the Ecuadorian Andes Mountains. Significant correlations between blood lead levels and either DPOAE results or pure-tone thresholds (1000 to 8000 Hz) were not found (*p* > 0.05).

Alvarenga et al. [76] used pure-tone audiometry, ABR and transient-evoked OAE (TEOAE) to investigate the association between these tests and low but long-term lead exposure in 130 children living near a battery factory. Blood lead levels were monitored for 35.5 months and audiological evaluations were carried out once. Results indicated normal hearing thresholds, normal amplitudes for TEOAE and no association (no *p*-value reported) between ABR results (absolute latencies for waves I, III and V as well as I–III, I–V and III–V IPL) and cumulative blood lead level. 

Counter et al. [77] investigated the ipsilateral and contralateral acoustic stapedius reflex (500, 1000 and 2000 Hz, and broadband noise) in a group of 117 children with chronic lead exposure in the Ecuadorian Andes. Acoustic reflex thresholds, amplitude growth and decay were investigated. Results showed no significant correlations between blood lead levels and acoustic reflex test results at any of the frequencies tested (500 Hz: *p* = 0.984; 1000 Hz: *p* = 0.426 Hz; 2000 Hz: *p* = 0.664).

Prenatal lead exposure and adverse auditory effects have also been investigated. Rothenberg et al. [78] reported an association (*p* = 0.0802) between higher maternal blood lead level at 20 weeks of pregnancy and increased ABR I–V and III–V IPL in one-month-old babies. In addition, Rothenberg et al. [79] investigated the possible association between prenatal and perinatal blood lead level and ABR results. Results showed significant correlations between maternal blood lead levels and ABR IPL. For example, the I–III IPL was increased with increasing maternal blood lead levels at 20 weeks (*p* = 0.001). In addition, a significant increase in I–V IPL was observed with increasing maternal blood lead levels at 12 weeks (*p* = 0.03). Rothenberg et al. [80] then followed up a group of more than 1000 children during 5–7 years after birth. The authors reported that maternal blood lead levels at 20 weeks of pregnancy was the only prenatal lead level significantly associated with ABR I–V (*p* = 0.008) and III–V IPL (*p* = 0.009), when controlling for confounders. Similarly, Geng et al. [81] reported that two-month-old infants with cord-blood lead concentrations above 2 g/dL did not present with differences (no *p*-value reported) in amplitudes for event-related potential components such as P2, P750 and late slow wave when using their mother’s voice versus strangers’ voices as eliciting stimuli. Contrarily, infants with cord-blood lead levels below 2 g/dL showed differences in amplitudes between both stimuli for the aforementioned responses (no *p*-value reported).

### 3.4. Manganese

Manganese is a natural component of air, water, soil and food [82]. Activities such as welding [83,84], smelting [85], industrial processes related to manganese based products [86], and the addition of methylcyclopentadienyl manganese tricarbonyl (MMT) to unleaded gasoline [87] all result in additional manganese contamination in the air [88]. However, this contamination is often composed of manganese as part of an organic compound, which makes determining the effects of manganese alone difficult [82]. In this literature review, only one study investigating the auditory effects in humans of manganese exposure was found.

Wennberg et al. [85] investigated a group of 30 male workers involved in steel smelting with manganese exposure along with 60 age-matched control workers without manganese exposure. Among other health outcomes the ABR and an event-related evoked potential (i.e., P300) were carried out. Results showed no significant differences between manganese-exposed and control workers for the ABR (absolute latencies for waves I, III and V, and I–III, I–V, and III–V IPL) and for the P300 latency (no *p*-values reported).

### 3.5. Mercury

Metallic or inorganic compounds containing mercury can be ingested by humans via inhalation or by drinking and using contaminated water for agriculture [89]. Man-made environments such as gold mines [90], industrial lamp factories [91] and other factories involving gold-mercury or silver-mercury amalgams are examples of sources of occupational exposure to mercury. In addition, mercury, being a stable amalgam when added to gold or silver, has been used as a dental filling [92]. These sources of contamination are not exclusive, as many others are a threat to human health as well (see Park et al. [89] for a review). Table 4 provides a summary of the studies investigating the auditory effects of mercury in children and adults. 

Discalzi et al. [61] conducted a study investigating ABR responses in workers exposed to mercury. The study group was composed of eight workers (6 males and 2 females) exposed to mercury with a mean age of 38.6 ± 13.2 years and a mean duration of mercury exposure of 11.7 ± 8.0 years. As a control group, eight age- and gender-matched subjects without self-reported history of exposure to any neurotoxic substances were selected. All participants had normal hearing thresholds (≤25 dB HL from 500 to 4000 Hz) and no history of neurologic, psychiatric, otologic or any other chronic diseases. Mean level of urinary mercury for the study group was below neurotoxic levels determined by the World Health Organization. ABR peak latencies for waves I and V were measured using click-type stimuli with alternated polarity, a rate of 11/s and an intensity of 100 dB SPLpe. I–V IPL results showed a significant difference (*p* = 0.033) between exposed and control groups. No correlation between duration of exposure and IPL in the study group was found (no *p*-value reported). Rothwell et al. [93] investigated the association between amalgam fillings and hearing thresholds. Subjects’ inclusion criteria were comprised of being female, nonsmoker, 40–45 years old, and presenting with subjective normal hearing, no family history of hearing loss, no ear surgery, no significant episodes of vertigo and normal otoscopy and tympanometry. A total of 39 subjects were selected as the study group. A qualified dentist collected data on the number of fillings, the component of the fillings (amalgam, porcelain or gold-filling), the number of drills and a filling score relating to surface area. Standard pure-tone thresholds (250, 500, 1000, 2000, 4000, and 8000 Hz) as well as ultra-high pure-tone thresholds (9, 10, 11.2, 12.5, 14, 15, and 16 kHz) were obtained. Results indicated a significant association between the filling score and hearing thresholds at 8 (*p* = 0.039), 11.2 (*p* = 0.044), 12.5 (*p* = 0.005), 14 (*p* < 0.001), and 16 kHz (*p* = 0.010). The hearing threshold at 14 kHz showed the strongest correlation (*p* < 0.001), where each additional amalgam filling was associated with a 2.4 dB increase in hearing threshold (95% CI 1.3–3.5 dB). 

Al-Batanony et al. [91] investigated the prevalence of hearing loss in 138 workers exposed to mercury in a fluorescent lamp factory in comparison to a control group of 151 subjects with no history of occupational exposure to mercury. Control group subjects were matched with exposed subjects for age, residence, income and level of education. Mercury-exposed subjects presented with a higher prevalence of hearing loss than non-exposed subjects (*p* = 0.03). In addition, the authors evaluated noise level exposure among mercury exposed subjects and found it to be below permissible levels in Egypt, which is 90 dB [94]. 

Different results were found by Shargorodsky et al. [25]. The authors found no association (*p* = 0.13) between blood mercury levels and hearing loss for either low-frequency PTA (500, 1000 and 2000 Hz) or high-frequency PTA (3000, 4000, 6000 and 8000 Hz) in 2535 participants selected from the 2005–2008 U.S. National Health and Nutrition Examination Survey. Similarly, Lille et al. [62] did not find abnormal ABR results for a group of nine patients either occupationally exposed to mercury or who had had accidental intoxication with this chemical. ABR results were compared with normative values and with a group of non-exposed control subjects (no specific results were provided in the paper).

Dutra et al. [95] investigated the central auditory function in adolescents exposed to mercury in Brazil. The study group was comprised of 21 (11 females and 10 males) adolescents who either worked in the burning of gold-mercury amalgams, the burning of gold in stores or who lived near these places. The control group was composed of 31 (17 females and 14 males) adolescents who did not have a history of mercury exposure. All participants had hearing thresholds within normal limits (i.e., ≤25 dB from 250 to 8000 Hz) and normal otoscopic and tympanometric results. The evaluation of the central auditory function included procedures evaluating sound localization, sequential memory for verbal and nonverbal stimuli, speech recognition in quiet, speech-in-noise as well as the frequency pattern, duration pattern, and staggered spondaic word tests. Results showed that mercury-exposed adolescents presented with significantly worse results than non-exposed adolescents for the sequential memory task of nonverbal stimuli (*p* = 0.001), and for the frequency pattern (right ear: *p* = 0.000; left ear: *p* = 0.000), duration pattern (right ear: *p* = 0.000; left ear: *p* = 0.000) and staggered spondaic word tests (right ear: *p* = 0.006; left ear: *p* = 0.005). The authors concluded that mercury has an adverse neurotoxic effect on the auditory system.

Counter et al. [96] investigated 40 people (21 children and 19 adults) exposed to mercury. A significant correlation between the hearing threshold at 3 kHz in the right ear and blood lead level in children was found (r = 0.54, *p* = 0.01). The ABR was conducted in a subsample of 19 children and adults. Normal ABR values for absolute latencies as well as IPL (I–III, III–V, and I–V) were found in all subjects. Correlations between left ear ABR results and blood mercury level were performed. No significant correlations between blood mercury level and absolute latencies for waves I (r = −0.028, *p* = 0.24), III (r = −0.23, *p* = 0.34) and V (r = −0.05, *p* = 0.83) and I–III (r = 0.45, *p* = 0.51), III–V (r = −0.10, *p* = 0.68) and I–V (r = 0.19, *p* = 0.42) IPL were found. 

### 3.6. Mixture of Heavy Metals

Factories relating to the use of mixtures of heavy metals are an important source of contamination, especially in non-occupational populations. This is because when these factories are shut down, accumulated heavy metals stay in the surrounding environment for many years [97]. Consequently, a mixture of these elements can be originated by historical events associated with the closedown of factories (natural or man-made) and can be linked to factories involved with the use of diverse heavy metals in their production [20,98]. Table 1 provides a summary for the studies discussed below. 

Araki et al. [98] investigated the possible association between occupational exposure to copper, lead and zinc and results for event related potentials in 22 male gunmetal foundry workers. Their tenure at the factory was between three and 18 years. The control group was composed of 14 male steel foundry workers who had no history of exposure to copper, lead or zinc, and no history of neurological disorders. As expected, exposed subjects presented with significantly higher copper, lead and zinc blood levels than non-exposed subjects. Two auditory late latency responses were obtained, a negative (N100) and positive (P300) one. Latencies for both responses were significantly longer in exposed subjects as compared to non-exposed subjects (no *p*-value reported). N100 latency among exposed subjects was significantly correlated with age (*p* < 0.05) but not with blood or urinary heavy metal levels (*p* > 0.05). In addition, P300 latency in the exposed group was significantly correlated with blood and urinary lead levels (*p* < 0.05). 

Saunders et al. [20] investigated the effects of exposure to a mixture of heavy metals on hearing thresholds and DPOAE in subjects living in Bonanza, Nicaragua. Fifty-nine gold mining workers were selected. All subjects presented with normal results for otoscopy and tympanometry. A questionnaire collecting demographics, fish consumption, drinking water source, subjective hearing ability, subjective noise exposure and occupation was carried out. In addition, pure-tone thresholds and DPOAE were obtained. As a biometric marker, nail clippings were collected to obtain the levels for 17 metals (i.e., aluminum, vanadium, chromium, manganese, iron, cobalt, nickel, copper, zinc, arsenic, selenium, molybdenum, cadmium, tin, mercury, lead, and uranium). Finally, subjects were categorized as either with high noise exposure (those ones who worked 40 h or more per week) or with low noise exposure (those ones who worked less than 40 h per week). Significant correlations were found between pure-tone thresholds and age at 2000, 4000 and 6000 Hz; however no correlation between reported noise exposure levels and DPOAE amplitudes was found (no *p*-value reported). In addition, nail arsenic levels were significantly correlated with DPOAE amplitudes at 2000 Hz (*p* = 0.04) for the low-noise group. A significant association was reported between nail lead levels and DPOAE amplitudes at 3000 (*p* = 0.01) and 4000 Hz (*p* = 0.03) as well as with the mean DPOAE amplitude (*p* = 0.03), which was also found among subjects with low noise level exposure. A correlation between nail manganese (*p* = 0.03) as well as nail aluminum (*p* = 0.04) levels and DPOAE amplitudes at 3000 Hz was only found in the low noise exposure group. 

Chuang et al. [99] investigated the possible association between exposure to a mixture of heavy metals (i.e., lead, manganese, arsenic and selenium) and hearing thresholds. Pure-tone thresholds (500, 1000, 2000, 3000, 4000 and 6000 Hz) and heavy metal levels in blood were obtained. Two groups of subjects were selected, 121 factory workers with hearing thresholds worse than 25 dB HL (study group) and 173 factory workers with hearing thresholds equal or better than 25 dB HL (control group). Confounding variables such as noise exposure and age were also investigated. Regression models showed that hearing thresholds were best predicted by lead (*p* < 0.001) and selenium (*p* = 0.033) blood levels along with age (*p* < 0.001). A dose–response relationship between blood lead levels and hearing thresholds was found after controlling for noise exposure and age (*p* < 0.001).

### 3.7. Heavy Metals and Noise

Hwang et al. [35] investigated a group of 412 steel plant workers who were exposed to noise and lead. Results showed that workers with high blood lead levels (equal to or above 7 μg/dL) exposed to noise levels equal to or above 85 dBA presented with significantly worse hearing thresholds at 3000, 4000 and 6000 Hz (*p* < 0.05) than workers with moderate and low blood lead levels who were co-exposed to high noise levels (equal to or above 85 dB). Similarly, workers with high blood lead levels exposed to noise levels between 80 and 85 dBA presented with significantly worse hearing thresholds at 4000 and 6000 Hz (*p* < 0.05) than workers exposed to the same noise levels but with moderate or low blood lead levels. No significant differences among workers with low, moderate and high blood lead levels who were exposed to noise levels below 80 dBA were found, perhaps because of the small sample size in these categories. Thus, the authors found an interaction between co-exposure to lead and noise at moderate and high levels. 

Wu et al. [39] investigated a group of 339 workers from two battery-making factories in Taiwan. Lead and noise exposure levels were determined for all workers. Hearing ability was measured as the hearing threshold at 4000 Hz in the worse ear. Multivariate analysis showed a significant association between a high, long-term lead exposure index and decreased hearing ability (*p* < 0.01). However, the authors did not find a significant interaction between noise exposure level and short- or long-term lead exposure on the hearing ability (no *p*-value reported). 

Finally, Counter and Buchanan [57] investigated 30 ceramic-glazing workers (15 men and 15 women) exposed to lead and noise. Pure-tone audiometry results showed that 60% of men and 20% of women presented with high frequency (3000–8000 Hz) hearing loss. No significant association between blood lead levels and hearing thresholds (3000–8000 Hz) was found (no *p*-value reported). The authors attributed the observed prevalence of hearing loss mainly to noise exposure. This was because of the difference for hearing loss prevalence between men and women and that female workers were typically not exposed to noise related to lead glazing operations. However, it should be noted that noise exposure levels were not reported. The authors pointed out that a possible enhancement of hearing loss due to the combination of lead and occupational noise exposure cannot be completely ruled out.

## 4. Discussion

In this literature review, a total of 49 studies investigating the adverse effects of heavy metal exposure on the human auditory system were found. Three studies were identified as addressing co-exposure to noise and heavy metals in humans. Studies investigating the auditory effects of arsenic, cadmium, lead, manganese, mercury and mixtures of heavy metals were found. No studies investigating the auditory effects in humans of chromium, cobalt, copper, nickel or zinc were found. 

Most of the studies found investigated the effects of lead exposure on the auditory system using different hearing outcomes. Some studies suggest that lead may induce higher hearing thresholds in adult populations either occupationally (e.g., [35,39,55,56,57]) or non-occupationally exposed to this chemical (e.g., [25,31,58]). In addition, lead exposed workers have been found to present with longer ABR IPL than non-exposed controls [59,60,61] suggesting that this chemical may adversely affect the central auditory pathways at the brainstem level. Similar results have been found in non-occupational populations of adults with a history of lead exposure [63]. In pediatric populations, it has been found that lead exposure may mainly affect ABR absolute latencies (e.g., [70]) and pure-tone thresholds (e.g., [5,66]), suggesting lead-induced sensorineural hearing loss. In addition, it has been shown that high prenatal blood levels may adversely affect the development of the auditory system (e.g., [78,79,80,81]).

Evidence about arsenic suggests that this heavy metal may be associated with higher hearing thresholds in children (e.g., [22]) and in adults (e.g., [18]). For cadmium, studies have found an association between this chemical and higher hearing thresholds (e.g., [25,31]) as well adverse central auditory effects [30]. Regarding mercury, it has been shown that this metal may affect the ABR IPL (e.g., [61]) and hearing thresholds especially at high frequencies (e.g., [91,93,96]). Finally, interactions between co-exposure to lead and noise at moderate and high levels have been found [35]. 

In summary, human studies suggest that exposure to heavy metals may adversely affect sound detection abilities as well as the central auditory system. Little evidence about dose–response relationships is available and a synergistic effect between heavy metal and noise exposure is not available from human data. 

## 5. Clinical Implications and Need for Further Research

Some of the studies have found that humans exposed to heavy metals such as lead and mercury may present with worse pure-tone thresholds and prolonged ABR IPL as compared with non-exposed populations. Thus, in the clinical setting, audiologists should include questions regarding heavy metal exposure when taking patients’ clinical history. In addition, taking these findings into account, clinicians evaluating patients with known history of heavy metal exposure should consider auditory signs beyond sound detection difficulties. Tests to explore the central auditory pathway should be included when this patient population is evaluated. ABR would be the most appropriate test, as most of the current evidence regarding central auditory effects induced by heavy metals is available from studies using this technique. If clinicians do not have access to ABR equipment, then an assessment of central auditory functions using behavioral techniques may be used.

In the workplace, hearing conservation programs should include all workers exposed to heavy metals regardless of their noise exposure levels. Baseline assessments should be carried out in workers commencing to perform duties in environments contaminated with heavy metals. Thereafter, periodic examinations should be conducted with the aim to determine the existence of changes in test results. Ideally, for such purposes, pure-tone audiometry along with ABR should be used. When the latter technique is not available, behavioral central auditory processing tests may be used. 

Further research about the adverse auditory effects of heavy metals in humans is needed. Future studies should determine the signs and symptoms of auditory dysfunctions induced by heavy metal exposure. In addition, a possible synergistic effect of co-exposure to noise and heavy metals should be investigated in humans, as limited evidence about the interaction between noise and heavy metals exists. From a clinical point of view, there is a need for an audiological test battery to evaluate patients with a history of heavy metal exposure. Thus, further research should be conducted with the aim to determine which tests beyond pure-tone audiometry are more sensitive to detect central auditory signs induced by heavy metal exposure. These tests should be easily accessible to clinicians and professionals relating to hearing conservation programs. Finally, there is limited understanding about the audiometric frequencies mainly affected by heavy metal exposure. Further research in humans should consider this limitation in order to determine whether hearing losses induced by heavy metal exposure can be differentiated from noise-induced hearing loss. 

## 6. Conclusions

In this literature review, a total of 49 studies investigating the adverse effects of heavy metal exposure on the human auditory system were found. Studies about the auditory effects of arsenic, cadmium, lead, manganese, mercury and mixtures of heavy metals were found. No studies about the auditory effects in humans of chromium, cobalt, copper, nickel or zinc were found. Lead-exposed human subjects were found to present with higher (i.e., worse) hearing thresholds than non-exposed controls. Also, lead-exposed human subjects have been found to present with longer ABR IPL than non-exposed controls suggesting that this chemical may adversely affect the brainstem auditory pathways. Similar results have been found in pediatric populations exposed to lead. Interactions between co-exposure to lead and noise at moderate and high levels have been found. In addition, it has been found that arsenic may be associated with higher hearing thresholds in adults and children. For cadmium and mercury, studies have found an association between exposure to these agents and higher hearing thresholds as well adverse central auditory effects. Little evidence about dose-response relationships is available and a synergistic effect between heavy metal and noise exposure is not available from human data. It is concluded that further evidence about the adverse auditory effects of heavy metals is required. Hearing health-care professionals should be aware of the possible deleterious effects of heavy metals on the auditory system and a comprehensive audiological assessment should be considered when suspecting auditory dysfunction induced by heavy metal exposure.

## Figures and Tables

**Table 1:** Summary of studies investigating the effects of arsenic, cadmium, manganese and mixture of heavy metals exposure in adults and children.

Reference	Heavy Metal(s)	Study Design	Population	Procedures	Results
Guo et al. (2007) [18]	As	Cross-sectional with a control group	Adults	Hearing loss prevalence	Higher prevalence of hearing loss
Bencko and Symon (1977) [22]	As	Cross-sectional with a control group	Children	Pure-tone audiometry	Arsenic-exposed children presented with significantly higher (worse) pure-tone thresholds than non-exposed children for 125, 250 and 8000 Hz
Milham (1977) [23]	As	Descriptive	Children	Hearing screening	No higher prevalence of failed hearing screening
Supapong and Sriratanabun (2005) [24]	As	Cross-sectional with a control group	Adults	Auditory brainstem response (ABR)	No significant differences between groups found
Shargorodsky et al. (2011) [25]	As	Cross-sectional without a control group	Adults	Pure-tone audiometry	No association found
Shargorodsky et al. (2011) [25]	Cd	Cross-sectional without a control group	Adults	Pure-tone audiometry	Highest cadmium urinary levels quartile had higher odds of an increased low-frequency pure tone average (500, 1000 and 2000 Hz)
Thatcher et al. (1984) [30]	Cd	Cross-sectional without a control group	Children	Long latency AEP	Association between cadmium level in hair and amplitude of one of the components of the AEP
Choi et al. (2012) [31]	Cd	Cross-sectional without a control group	Adults	Pure-tone audiometry	Increase in PTA by 13.8% between highest and lowest quintile levels of cadmium, adjusted for noise and other major risk factors for hearing loss.
Wennberg et al. (1991) [85]	Mn	Cross-sectional with a control group	Adults	ABR and P300	No significant differences found
Araki et al. (1992) [98]	Cu, Pb and Zn	Cross-sectional with a control group	Adults	AEP- N100 and P300	P300 latency in the exposed group was significantly correlated with blood and urinary levels of lead
Chuang et al. (2007) [99]	Pb (Se, As, Mn)	Cross-sectional with a control group	Adults	Pure-tone audiometry	A dose–response association was found between blood lead levels and average hearing thresholds
Saunders et al. (2013) [20]	Al	Cross-sectional without a control group	Adults	Pure-tone audiometry and DPOAE	Correlation with decreased DPOAE amplitudes at 3000 Hz in subjects with low noise exposure levels. No correlation with pure-tone thresholds
	As				Correlation with decreased DPOAE amplitudes at 2000 Hz in subjects with low noise exposure levels. No correlation with pure-tone thresholds
	Hg				No correlation found
	Mn				Correlation with decreased DPOAEs amplitude at 3000 Hz in subjects with low noise exposure levels. No association with pure-tone thresholds
	Pb				Correlation with DPOAE amplitude at 3000 Hz, 4000 Hz, and the mean DPOAE amplitude in subjects with low noise exposure levels. No correlation with pure-tone thresholds

**Table 2:** Summary of studies investigating the effect of lead exposure in adulthood.

Reference	Study Design	Procedures	Results
*Hearing thresholds and occupational lead exposure*			
Choi et al. (2012) [31]	Cross-sectional without a control group	Pure-tone audiometry	The highest quintiles of lead blood levels were associated with increases in PTA compared to the lowest quintiles (after adjusting for sociodemographic and clinical risk factors and exposure to occupational and non-occupational noise).
Hwang et al. (2009) [35]	Cross-sectional without a control group	Pure-tone audiometry	Association between lead in blood and hearing thresholds at most of the audiometric frequencies. A logistic regression model, adjusted for age and noise exposure level, showed that blood levels above 7 µg/dL were significantly associated with hearing loss at 3000 and 8000 Hz.
Wu et al. (2000) [39]	Cross-sectional without a control group	Pure-tone audiometry	Association between long-term lead exposure and higher pure-tone threshold at 4000 Hz in the worse ear. No association for short-term lead exposure. No interaction with noise and exposure level was found.
Baloh et al. (1979) [54]	Cross-sectional with a control group	Hearing loss prevalence	No significant association found.
Farahat et al. (1997) [55]	Cross-sectional with a control group	Pure-tone audiometry	Lead-exposed workers presented with higher (worse) hearing thresholds for the 1000–8000 Hz range than non-exposed workers. Hearing threshold at 8000 Hz was the frequency most significantly affected by lead exposure. Hearing thresholds were found to correlate significantly to blood lead levels and years of lead exposure.
Forst et al. (1997) [56]	Cross-sectional without a control group	Pure-tone audiometry	Significant correlation between blood lead level and hearing threshold only at 4000 Hz.
Counter and Buchanan (2002) [57]	Cross-sectional without a control group	Pure-tone audiometry and ABR	Mean pure-tone thresholds from 2000 to 8000 Hz showed sensorineural hearing loss among exposed male subjects. Bilateral ABR on workers with elevated blood lead levels showed delayed wave latencies.
*Hearing thresholds and non-occupational lead exposure in adulthood*			
Shargorodsky et al. (2011) [25]	Cross-sectional without a control group	Pure-tone audiometry	Significant association between the quartile with the highest level of lead exposure and a higher high-frequency PTA.
Park et al. (2010) [58]	Cross-sectional without a control group	Pure-tone audiometry	Trabecular bone lead levels were significantly associated with poorer hearing thresholds (at 2000, 3000, 4000, 6000 and 8000 Hz), PTA (mean of 500, 1000, 2000 and 4000 Hz) and odds of hearing loss. Significant positive longitudinal association between cortical bone lead levels and the rate of change in hearing thresholds at 1000, 2000 and 8000 Hz, as well as with PTA.
*Auditory brainstem response and occupational lead exposure*			
Murata et al. (1995) [37]	Cross-sectional with a control group	ABR	No significant differences between groups found.
Yokoyama et al. (2002) [38]	Cross-sectional with a control group	ABR	No significant differences between groups found.
Discalzi et al. (1992) [59]	Cross-sectional without a control group	ABR	Lead exposed workers had significantly longer I–V IPL than non-exposed subjects.
Bleecker et al. (2003) [60]	Cross-sectional without a control group	ABR	Significant correlation between ABR wave I latency and blood lead levels. Significant association between working-lifetime weighted average blood lead and ABR wave III latency. Association between abnormal ABR waves I absolute latency and I–V IPL and lead exposure levels.
Discalzi et al *.* (1993) [61]	Cross-sectional with a control group	ABR	Lead-exposed subjects presented significantly longer I–V IPL than an age- and gender-matched control group.
*Auditory brainstem response and non-occupational lead exposure in adulthood*			
Holdstein et al. (1986) [63]	Cross-sectional without a control group	ABR	Significant association between higher blood lead levels and longer I–III IPL. Significant correlations between blood lead levels and III–V IPL.

**Table 3:** Summary of studies investigating the effect of lead exposure in childhood.

Reference	Study Design	Procedures	Results
Buchanan et al. (2011) [2]	Cross-sectional cohort without a control group	Pure-tone audiometry and DPOAE	No association found.
Osman et al. (1999) [5]	Cross-sectional without a control group	Pure-tone audiometry	Association between blood lead levels and hearing thresholds. Children with the highest blood lead levels presented with a significantly increased latency of ABR wave I (adjusted for age) when compared to children with lowest blood lead levels.
Otto et al. (1985) [64]	Cross-sectional without a control group	ABR	Association between blood lead levels and absolute wave latencies for waves III and V.
Abdel Rasoul et al. (2012) [65]	Cross-sectional without a control group	Pure-tone audiometry	Blood lead levels were significantly correlated with pure-tone thresholds.
Schwartz and Otto (1987) [66]	Cross-sectional study without a control group	Pure-tone audiometry	Blood lead levels were significantly associated with increased right and left hearing thresholds at 500, 1000, 2000 and 4000 Hz.
Schwartz and Otto (1991) [67]	Cross-sectional study without a control group	Pure-tone audiometry	Significant association between blood lead levels and pure-tone thresholds at 500 Hz, 1000 Hz, 2000 Hz, and 4000 Hz.
Kamel et al. (2003) [68]	Cross-sectional study without a control group	Pure-tone audiometry	Significant correlation between blood lead level and PTA.
Baumann et al. (1987) [69]	Cross-sectional study without a control group	Long latency AEP	Significant association between blood lead level and the positive peak of the long latency AEP.
Zou et al. (2003) [70]	Cross-sectional without a control group	ABR	Significant association between high blood lead levels and longer peak-latencies for I, III and V. Significant positive correlations between peak-latencies for waves I, III and V in both ears and blood lead levels.
Counter et al. (1997) [72]	Cross-sectional with a control group	Pure-tone audiometry and ABR	No association found.
Counter (2002) [73]		Pure-tone audiometry and ABR	No association found.
Counter et al. (2012) [74]	Cross-sectional without a control group	ABR	No significant association between blood lead levels and ABR wave latencies.
Buchanan et al. (1999) [75]	Cross-sectional without a control group	Pure-tone audiometry and DPOAE	No association found
Alvarenga et al. (2015) [76]	Cross-sectional cohort without a control group	pure-tone audiometry, ABR and TEOAE	No association found.
Counter et al. (2011) [77]	Cross-sectional without a control group	Acoustic reflex thresholds, amplitude growth and decay	No significant correlations between blood lead levels and various acoustic reflex tests at any of the frequencies tested.
Rothenberg et al. (1995) [78]	Repeated measures without a control group	ABR	Association between higher maternal blood lead level at 20 weeks of pregnancy and increased ABR I–V and III–V IPL in 1-month-old babies.
Rothenberg et al. (2000) [80]	Cohort without a control group	ABR	Maternal blood lead levels at 20 weeks of pregnancy significantly associated with ABR I–V and III–V IPL in 5 year-old children.
Geng et al. (2014) [81]	Cross-sectional with a control group	ABR	Infants with cord-blood lead concentrations above 2 µg/dL did not present differences in amplitudes for event-related potential (P2, P750) and late slow wave when using their mother’s voice versus strangers’ voices as eliciting stimuli as opposed to infants with cord-blood lead concentrations below 2 µg/dL.

**Table 4:** Summary of studies investigating the effects of mercury exposure in children and adults.

Reference	Study Design	Population	Procedures	Results
Shargorodsky et al. (2011) [25]	Cross-sectional without a control group	Adults	Pure-tone audiometry	No association found.
Discalzi et al. (1993) [61]	Cross-sectional with a control group	Adults	ABR	Workers exposed to mercury had significantly increased I–V IPL compared to an age- and gender-matched non-exposed control group.
Al-Batanony et al. (2013) [91]	Cross-sectional with a control group	Adults	Pure-tone audiometry	Significant difference in the prevalence of hearing loss between workers exposed to mercury and the non-exposed control group.
Rothwell and Boyd (2008) [93]	Cross-sectional without a control group	Adults	Pure-tone audiometry	The number of dental amalgam fillings by surface area had a significant association with hearing thresholds at 8, 11.2, 12.5, 14, and 16 kHz.
Dutra et al. (2010) [95]	Cross-sectional with a control group	Adolescents	Central Auditory processing	Significant difference between groups for the results of sequential memory of nonverbal stimuli. Significant difference for temporal frequency, duration pattern, and staggered spondaic word tests. No significant difference between groups for the sequential memory of verbal stimuli and sound localization. No significant difference for speech test with competitive white noise.
Counter et al. (1998) [96]	Cross-sectional without a control group	Children and adults	Pure-tone audiometry and ABR	Significant correlation between the hearing threshold at 3 kHz in the right ear and blood lead level in children. No effect on ABR results found.

## Abbreviations

ABR	Auditory brainstem response
AEP	Auditory evoked potential
CI	Confidence interval
DPOAE	Distortion product otoacoustic emission
IPL	Inter-peak latency (for ABR)
OAE	Otoacoustic emission
OR	Odds ratio
PTA	Pure-tone average
TEOAE	Transient evoked otoacoustic emission
